# Notes and News

**Published:** 1890-06-14

**Authors:** 


### Notes and News.

Collected and Communicated.

The address delivered by Mr. Jonathan Hutchinson, President of the Royal College of Surgeons, to which we call attention elsewhere, is so excellent that we have made arrangements to reprint it in pamphlet form for general distribution by hospital authorities. It is so admirably written that it will serve as an eloquent appeal on behalf of each and all the hospitals throughout the country, the authorities of which are wise enough to utilize this great opportunity for adding to their funds. Chairmen of committees or secretaries can procure a specimen copy on application to the publisher of The Hospital, 140, Strand, W.C., together with the terms per thousand, or per five thousand, or per ten thousand, on which we are prepared to supply it, space being allowed for any statement on behalf of particular institutions which the authorities may desire to aid. We believe if this opportunity for widely circulating Mr. Hutchinson's address be promptly taken it will result in a larger sum being given to the hospitals than has yet been obtained from voluntary sources.

The ladies were very numerous at the festival dinner in aid of the Royal Blind Pension Society, and the secretary took advantage of the occasion to state that his experience went to prove that the presence of ladies went to swell the subscription list, and not to decrease it, as has been stated by some. Also the Marquis of Lorne, who presided, said he had been recently reading the life of Lord Melbourne, in which he had noticed the monstrous assertion, since repeated, that it was inadvisable to have ladies at a charitable banquet. He dissented altogether from this, for his experience had been that whenever anything good was being done, the old proverb of "cherchez la femme" was well applied. He made an earnest appeal on behalf of the charity, in response to which subscriptions to the amount of £1,765 were announced.

A public meeting followed at the Mansion House, Mr. Alderman and Sheriff Knill presiding in the absence of the Lord Mayor through ill-health, at which it was decided to make an appeal to the public for a sum of £3,000 urgently required for the present needs of the hospital. Some £2,000 of this debt had been caused by the necessity for providing further accommodation at the hospital, which necessity had been met to a certain extent by taking in an adjoining house in Hackney Road. The secretary (Mr. Arthur Nixon) announced subscriptions to the special fund amounting to £390, which included a cheque of £100 from the president (Mr. J. G. Barclay).

Lord F. J. Fitzroy presided on Friday at the 22nd annual meeting of the North-Eastern Hospital for Children, Hackney Road, which was held at the City offices, 27, Clements Lane, E.C. From the annual report we find that the number of cases treated during the past year was 531 in-patients, and 14,550 new cases of out-patients, with 51,087 attendances. The average annual number of out-patients for the last ten years was over 13,500, and of attendances over 46,500.

The Convalescent Home at Heylesbury, Wilts, is open from May 1st to November 1st, and during these six months it treated 44 patients at a cost of £105. This sanatorium is for women and girls who are recommended by subscribers, but patients are also admitted on payment of 8s. a-week. The Lady Superintendent, Miss Elling, does much for the comfort of the inmates. The Sydney Cottage Hospital issues its eighth annual report, telling of 29 patients treated and a small balance carried forward to next year.

We beg to call the attention of the editor of our excellent contemporary, the *Philanthropist*, to a correspondent of the "Man of Ross," who, on page 89 of the June number of that paper, has quoted bodily from an article on "The Christian Origin of Hospitals," which appeared in The Hospital on February 8, page 290. That article went to show what its title implies, that there were sound reasons for assuming the Christian origin of hospitals. The amusing part is that our contemporary's correspondent winds up with a quotation from another place, the source of which, though not mentioned, may be recognised, aiming to prove that hospitals did not originate with Christianity. It is interesting and instructive to note how copy is sometimes manufactured in these days of many journals. It is a compliment, of course, to be quoted, but not without acknowledgment.

Among those present at the annual meeting of the Hospitals Association at the Middlesex Hospital, on Wednesday, June 4th, were: Dr. J. S. Bristowe (the president) in the chair, Dr. T. Gilbart-Smith, Mr. H. Howgrave Graham (secretary, Hospital for Epilepsy), Dr. Haward, Mr. W. H. Hughes (secretary, Cancer Hospital), Surgeon-Major J. Ince, Mr. E. Murray Ind (chairman, London Hospital), Rev. J. Mahomed (chaplain, London Hospital), Rev. J. Marshall (Westminster Hospital), Mr. F. Clare Melhado (secretary, Middlesex Hospital), Mr. P. Michelli (secretary, Seamen's Hospital), Lieut.-Colonel E. Montefiore, Sir William Moore, K.C.I.E., Mr. W. J. Nixon (house governor, London Hospital), Mr. Thomas Ryan (secretary, St. Mary's Hospital), Dr. J. C. Steele (superintendent, Guy's Hospital), Mr. Joseph White, F.R.C.S. (surgeon, Nottingham General Hospital), Mr. Percy Wigram, Mr. Keith D. Young, F.R.I.B.A.

The new Infirmary at Liverpool begins to look an imposing structure, and should soon be ready for the opening ceremony. As Mr. Waterhouse is the architect, there is no necessity to dwell on the grand and picturesque outlines of the building. The wards are arranged in six blocks, three on either side of this immense corridor, and they will contain 290 beds. The wards on the first floor are for females, and those on the second floor for males. The oblong wards to the south-east and south-west, which, on the second floor, take 32 beds each, are, on the first floor, curtailed to give space for small pay wards and rooms for sick nurses, in the south-east and in the south-west block for operating rooms in connection with the obstetric ward, to be known as the Thornton ward, in honour of a lady who some time ago generously aided the funds of the institution to the extent of £10,000. The latter block has two small theatres attached, while all the wards have a scullery, a Sister's room, a doctor's room, a separate room for two beds, and a dining-room for convalescents. All the details have been carefully carried out; the cornices are rounded, every material used is fireproof, and the kitchen is fitted with all the most improved appliances. Let us hope Liverpool will be right proud of its new infirmary, and maintain it munificently, as it deserves.

The following vacancies are announced this week, the amount of the salary in each case being given after the name of the institution :—

**Resident Medical Officers.**

Jarrow-on-Tyne Memorial Hospital, House Surgeon—£150, board, etc.

Royal Free Hospital, Junior Medical Officer—board, etc.

The Royal General Dispensary, E.C., Medical Officer.

Bristol General Hospital, House Surgeon—£120, board, etc.

Stockport Infirmary, House Surgeon—£100, board, etc.

Carmarthen Infirmary, House Surgeon—£100, board, etc.

Derbyshire General Infirmary, Assistant House Surgeon—board, etc.

Hull Royal Infirmary, Assistant House Surgeon—£70, board, etc.

Derby Friendly Societies, Senior Medical Officer—£280, rooms, etc.

Luton Friendly Societies, Medical Officer—£180, rooms, etc.

**Non-Resident Medical Officers.**

Royal Westminster Ophthalmic Hospital, Clinical Assistants.

Royal Free Hospital, Assistant Surgeon.

Charing Cross Medical School, Demonstrator of Anatomy—£150.

The twenty-fourth anniversary of Dr. Barnardo's Homes was celebrated at the Royal Albert Hall on Wednesday evening, the Marquis of Lorne in the chair. The celebration consisted less of the dry details of an ordinary meeting than of a practical demonstration of the work of the homes The Ilford girls went through physical exercises, the little boys were drilled, and a choir of over 1,000 children sang till the building rang with their voices. Altogether the proceedings were animated and interesting.

Abbé Faure attends to the scaffold all the criminals executed on the square in front of the Roquette, and being used to the swift vengeance of the guillotine, the Abbé is shocked at the thought of execution by electricity. The worthy ecclesiastic says that he has the utmost sympathy for all scientific inventions, but he refuses to believe that French legislators will be so cruel as to protract by preparations which require a certain amount of time the sufferings of even murderers. The reason for employing electricity for executions is because the swiftness of the death is supposed to be merciful; but if, indeed, the preliminary preparations must be lengthy, the advantage is not so apparent.

The annual meeting of the governors and supporters of the East-end Mothers' Home was held lately at 30, Charles Street, by the kind permission of the Marchioness of Waterford. The Lord Bishop of London presided, and amongst those present were the Bishop of Bedford, the Marquis of Waterford, Sir William Marriott, M.P., and the Hon. Mrs. Stuart Wortley. The report of the Committee of Management, which was read by Mrs. Ashton Warner (the secretary), stated that during the financial year ending April 30th last, 168 patients had been registered for admission, making since the opening of the institution on December 6th, 1884, 716. The total number treated since the opening was 553. Extra expenses had been incurred in removing to the new home in Commercial Road, but the Committee felt very confident that in future years the expenditure would be relatively much reduced, on account of being able in the new premises to receive nearly double the number of patients. The Committee made an earnest appeal for further support in order that the beneficent work of the home might be continued and extended, and the heavy debt of £1,500 for the purchase of the property paid off. The Bishop of Bedford moved the adoption of the report, which he said was a satisfactory record of good work, and held out hope of still further benefits to the poor of the East of London in the future. The increased accommodation which was provided in the new home would, he hoped, encourage those who had the management of the charity not to enlarge their present hospital, but rather to increase the number of similar hospitals in that part of the East-end, because there was great need for such accommodation. There was no charity which did its work so satisfactorily as the one in whose interests they were met. Mr. Richard Woollcombe seconded the adoption of the report, which was agreed to.

